# Epigenomics‐guided precision oncology: Chromatin variants in prostate tumor evolution

**DOI:** 10.1002/ijc.35327

**Published:** 2025-01-23

**Authors:** Kira Furlano, Tina Keshavarzian, Nadine Biernath, Annika Fendler, Maria de Santis, Joachim Weischenfeldt, Mathieu Lupien

**Affiliations:** ^1^ Department of Urology Charité‐ Universitätsmedizin Berlin Berlin Germany; ^2^ Princess Margaret Cancer Centre University Health Network Toronto Canada; ^3^ Department of Medical Biophysics University of Toronto Toronto Canada; ^4^ Department of Urology Medical University of Vienna Vienna Austria; ^5^ Biotech Research & Innovation Centre (BRIC), The Finsen Laboratory, Rigshospitalet University of Copenhagen Copenhagen Denmark; ^6^ Ontario Institute for Cancer Research Toronto Ontario Canada

**Keywords:** chromatin variants, epigenomics, precision oncology, prostate cancer, tumor evolution

## Abstract

Prostate cancer is a common malignancy that in 5%–30% leads to treatment‐resistant and highly aggressive disease. Metastasis‐potential and treatment‐resistance is thought to rely on increased plasticity of the cancer cells—a mechanism whereby cancer cells alter their identity to adapt to changing environments or therapeutic pressures to create cellular heterogeneity. To understand the molecular basis of this plasticity, genomic studies have uncovered genetic variants to capture clonal heterogeneity of primary tumors and metastases. As cellular plasticity is largely driven by non‐genetic events, complementary studies in cancer epigenomics are now being conducted to identify *chromatin variants*. These variants, defined as genomic loci in cancer cells that show changes in chromatin state due to the loss or gain of epigenomic marks, inclusive of histone post‐translational modifications, DNA methylation and histone variants, are considered the fundamental units of epigenomic heterogeneity. In prostate cancer chromatin variants hold the promise of guiding the new era of precision oncology. In this review, we explore the role of epigenomic heterogeneity in prostate cancer, focusing on how chromatin variants contribute to tumor evolution and therapy resistance. We therefore discuss their impact on cellular plasticity and stochastic events, highlighting the value of single‐cell sequencing and liquid biopsy epigenomic assays to uncover new therapeutic targets and biomarkers. Ultimately, this review aims to support a new era of precision oncology, utilizing insights from epigenomics to improve prostate cancer patient outcomes.

## Graphical abstract

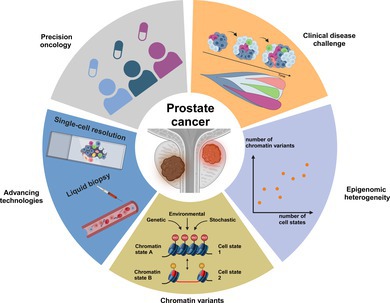

## 1 PROSTATE CANCER IS A MULTI‐FACETED DISEASE IN CLINICAL MANAGEMENT

**FIGURE 1:**
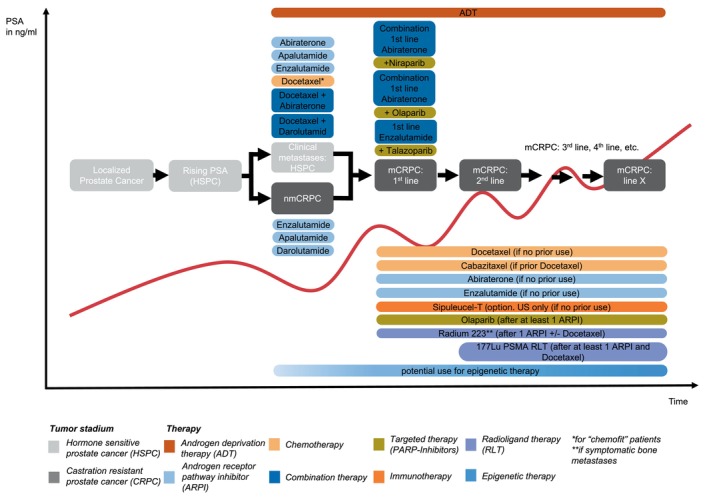
**Current clinical management and emerging therapeutic strategies in advanced prostate cancer**. Treatment landscape for advanced prostate cancer in 2024, illustrating the progression from localized disease to advanced metastatic castration‐resistant prostate cancer over time (x‐axis) dependent on the tumor burden (schematically outlined by PSA in ng/ml) (y‐axis). The treatment options are stratified, highlighting the sequential use of therapies and potential emerging treatments.

Prostate cancer is a leading cancer among men globally, posing a major public health challenge. Worldwide, the disease is expected to place increasing strain on health systems, with annual cases projected to rise from 1.4 million in 2020 to 2.9 million by 2040.^1^, ^2^ The disease typically originates from the glandular epithelium of the prostate and often progresses slowly during its early stages, remaining localized and asymptomatic for many years.^3^ However, in a subset of patients, it evolves into aggressive forms that metastasize to lymph nodes, bones, and other organs, contributing to substantial morbidity and mortality. Clinical management is stage‐dependent: Localized disease is typically managed with curative intent through surgical intervention, such as radical prostatectomy, or radiation therapy. In contrast, advanced disease often necessitates a multi‐modal approach. Systemic therapies play a pivotal role in the treatment of metastatic cases and include androgen deprivation therapy (ADT), next‐generation androgen receptor pathway inhibitors (ARPIs), and taxane‐based chemotherapy. Recently, non‐hormonal options, such as Poly (ADP‐ribose) polymerase (PARP) inhibitors, immunotherapies, and radioligand therapies, have expanded the therapeutic arsenal. In metastatic settings, these strategies are frequently combined to optimize treatment efficacy (see Figure 1). Prostate cancer presents as multiple entities due to its considerable heterogeneity.^4^, ^5^, ^6^, ^7^, ^8^, ^9^ This complexity creates challenges for precision oncology, as current methods of disease monitoring and treatment are often insufficient to effectively address the diverse nature of the disease.^10^

**FIGURE 2:**
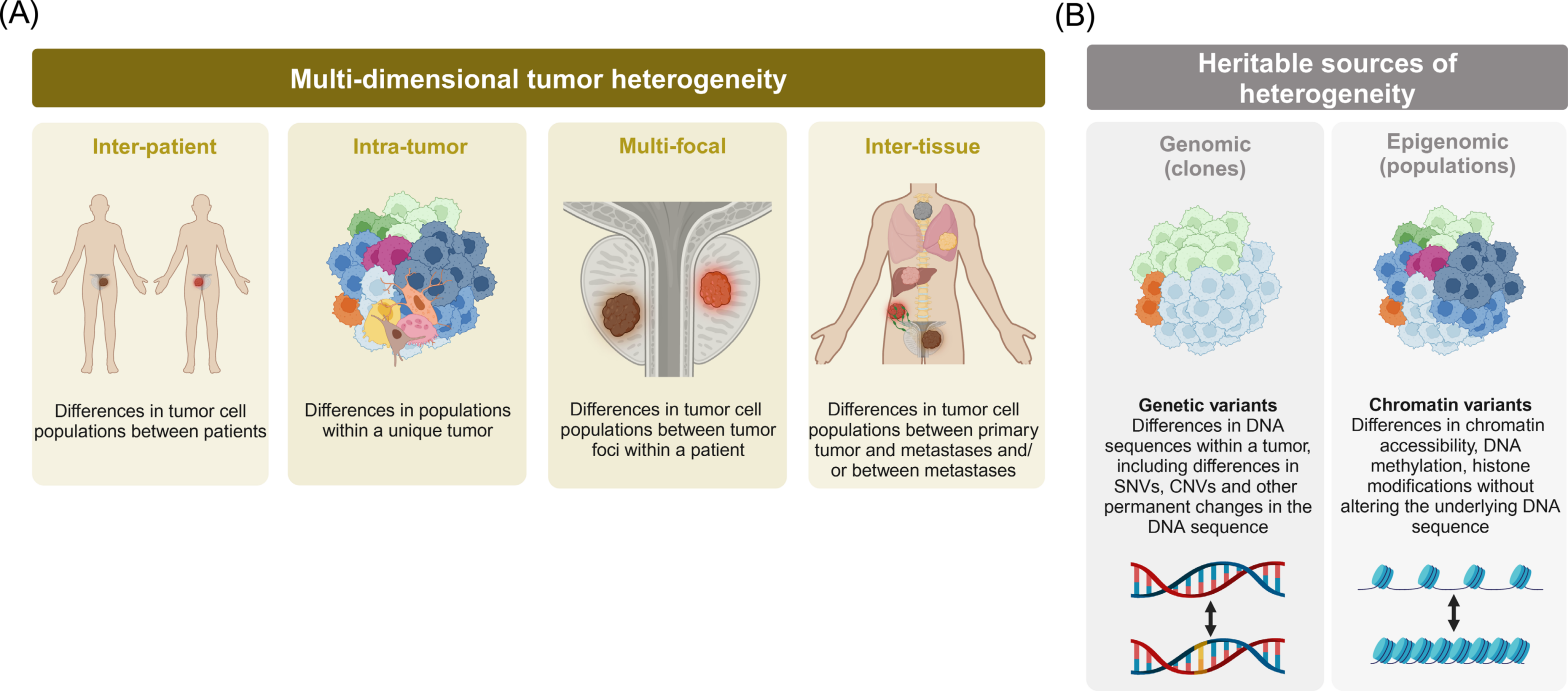
**Heterogeneity in prostate cancer**. (A) Multi‐dimensional heterogeneity of prostate cancer. Illustration of the various dimensions of tumor heterogeneity that contribute to the complexity of cancer diagnosis, prognosis, and treatment. Tumor heterogeneity can be categorized into four main types: Inter‐patient, intra‐tumor, multi‐focal and inter‐tissue heterogeneity. (B) Distinct roles of genetic and chromatin variants in tumor heterogeneity. These four dimensions can be assessed based on clones (genomics) or populations (epigenomics); genetic variants provide a stable, heritable basis for tumor diversity, while chromatin variants offer a flexible, potential reversible layer of regulation that allows for rapid adaptation. Understanding both processes is crucial for developing comprehensive cancer treatment strategies.

Prostate cancer displays significant heterogeneity across multiple dimensions. Genetically, intra‐tumor heterogeneity has been correlated with poorer prognosis, underscoring the complexity of tumor evolution and adaptation.^5^, ^11^, ^12^ Metastasis follows a complex pattern, with the primary tumor capable of initiating multiple waves of metastatic spread, either through monoclonal seeding or the dissemination of various subclones across different organs.^13^, ^14^, ^15^ Notably, locoregional lymph node metastasis frequently serves as a precursor for the dissemination to distant sites.^16^ The challenge lies in advancing our understanding of tumor heterogeneity beyond clonal characterization and in identifying and characterizing the heterogeneity of cellular populations to understand the genesis of prostate tumor evolution. Addressing this challenge also requires an understanding of tumor heterogeneity across multiple dimensions, inclusive of inter‐patient, intra‐tumor, multi‐focal and inter‐tissue tumor heterogeneity (see Figure 2A).

*Inter‐patient tumor heterogeneity* in prostate cancer has been extensively characterized through histo‐morphological and genotypic profiling in primary and metastatic settings.^6^, ^7^, ^8^, ^11^, ^17^, ^18^ This diversity is particularly pronounced in the most aggressive tumors, where it predicts patient outcomes and affects treatment responses, highlighting the challenges in effectively managing the disease.^19^ Large‐scale efforts on localized and metastatic prostate cancer have revealed molecular subtypes that differ at the genetic level despite having similar histological appearances.^7^, ^8^, ^17^, ^20^, ^21^, ^22^, ^23^ Therefore, mutations accounting for the cellular adaptations required for invasion and metastasis in high‐risk prostate cancer remain ill‐defined.^13^, ^24^

*Intra‐tumor heterogeneity* implies that alterations in the molecular characteristics of cancer cells and their surrounding microenvironment can occur in the composition of cellular populations.^11^, ^25^ The degree of variation between individual tumor cells, cellular populations, or geographically distinct tumor lesions found in a patient can be significant. These differences lead to the formation of cellular heterogeneity, which plays a critical role in determining a patient's tumor overall ability to evolve.^11^ In primary prostate tumors this intra‐tumor heterogeneity is well‐established, leading to variations in therapeutic responses across different tumor loci within a patient, even when they exhibit similar histological features.^5^, ^26^, ^27^, ^28^ For instance, localized prostate cancers that do not exhibit multiple sub‐populations within the tumor have significantly better outcomes compared to those with polyclonal tumors, even when considering pre‐treatment clinical factors such as prostate‐specific antigen (PSA) levels, Gleason score, and clinical T‐category.^29^ Zhang et al.^4^ identified by using spatially resolved single‐cell profiling two major types of genomically unstable cells—pseudo‐diploid and aneuploid—in localized early‐stage prostate cancer, with a specific sub‐population of pseudo‐diploid cells in tumor‐rich regions carrying sub‐chromosomal deletions that may indicate tumor‐initiating or driving cells.

In most patients, primary prostate cancer is characterized by multiple foci within the prostate,^30^ raising the question of whether these are numerous independent tumours, whether they have a common genetic ancestor or if another commonality is their basis. Histological assessment of *multi‐focal heterogeneity* within the prostate gland classifies prostate cancer patients according to morphological patterns defined by Gleason and the International Society of Uropathology (ISUP) grading systems. These patterns can differ significantly among different biopsy cores collected from the same prostate, often leading to discrepancies in grading.^9^, ^31^ Such variations are frequently observed and may be associated with different therapeutic responses.^4^, ^5^, ^6^, ^26^, ^28^, ^30^, ^32^, ^33^ Early multi‐regional tumor genome sequencing studies have shown that these foci often have limited overlap in detectable somatic mutations, such as substitutions and copy number alterations, indicating that they evolve independently, even though they may eventually present as a single lesion.^5^, ^12^

Despite the mult‐ifocal heterogeneity reported in primary prostate tumors, metastases represent an evolutionary bottleneck, and genetic studies point to only a few clones gaining metastatic potential.^16^, ^34^ Metastasis, a complex multi‐step process where cancer cells migrate from the primary site to distant organs, is responsible for the majority of cancer‐related deaths in prostate cancer.^35^ While metastasis and multiple waves of metastatic seeding from distinct subclones can occur,^13^, ^15^ genotypic profiling of metastatic tissues from the same patients (*inter‐tissue*) in a hormone‐sensitive setting reveals similarities across metastatic sites and resemblance to treatment‐resistant distal metastases.^14^, ^16^ Despite the often‐shared clonal origin of prostate cancer metastases, heterogeneity is still pervasive, as the metastatic disease is characterized by selective clonal sweeps, reseeding from metastasis to either a second metastasis or the primary tumour (bed), the occurrence of distinct genomic alterations, and convergent evolution.^13^

Considering its diverse dimensions, understanding prostate tumor heterogeneity and its impact on tumor evolution is central to the design of precision oncology, which aims to tailor treatments based on individual patient tumor characteristics. Efforts to date have focused on genetic heterogeneity assessments to identify the heritable basis of tumor evolution.^5^, ^12^, ^17^, ^36^, ^37^ The term “heritable” emphasizes the investigation into which alterations in a tumor are consistently transmitted to future generations of cancer cells, as opposed to those that are temporary and reversible. However, cell fate determination is inherently encoded in epigenomic variation in normal tissues, a process hijacked by cancer cells. Hence, a new era of precision oncology requires expanding the genetic effort to other non‐genetic heritable drivers of cell fate determination.

## 2 CHROMATIN VARIANTS AS HERITABLE NON‐GENETIC DRIVERS OF EPIGENOMIC HETEROGENEITY

As normal cells undergo fate commitment, they acquire heritable changes in their chromatin that generates *chromatin variants*.^38^, ^39^ Chromatin variants, the fundamental units of epigenomic variation, are genomic loci that exhibit distinct chromatin states across cells with different phenotypes. Chromatin variants are characterized by differences in epigenomic marks over a series of nucleosomes and the DNA linker region, such as changes in the positioning of nucleosomes, their composition (core vs. histone variants), and changes in DNA methylation and histone post‐translational modifications.^40^ These chromatin variants enable the selective activation or repression of genes necessary for cell fate changes and stable cellular states. Similar to what is observed throughout development, mounting evidence supports a direct role for chromatin variants as a “non‐genetic” driver of cellular heterogeneity in cancer.^41^, ^42^ Because cellular division equally involves the replication of chromatin, inclusive of DNA and associated proteins, mainly histones, chromatin variants serve as heritable drivers of phenotypic diversity, across physiological^43^, ^44^ and pathological contexts.^45^, ^46^, ^47^ Unlike genetic variants, which can drive genetic clonal heterogeneity, chromatin variants drive epigenomic heterogeneity, which reflects the diversity of cellular states within a sample (see Figure 2B). Chromatin variants differ from genetic variants in being somewhat labile, serving as ideal targets for drug intervention.^26^, ^47^, ^48^, ^49^, ^50^, ^51^ Hence, by identifying and characterizing cancer‐specific chromatin variants, we can understand the heritable basis of cell states and devise strategies to hinder epigenomic heterogeneity in prostate cancer.

## 3 CHROMATIN VARIANTS ARISE FROM DIRECTED AND STOCHASTIC PROCESSES

**FIGURE 3:**
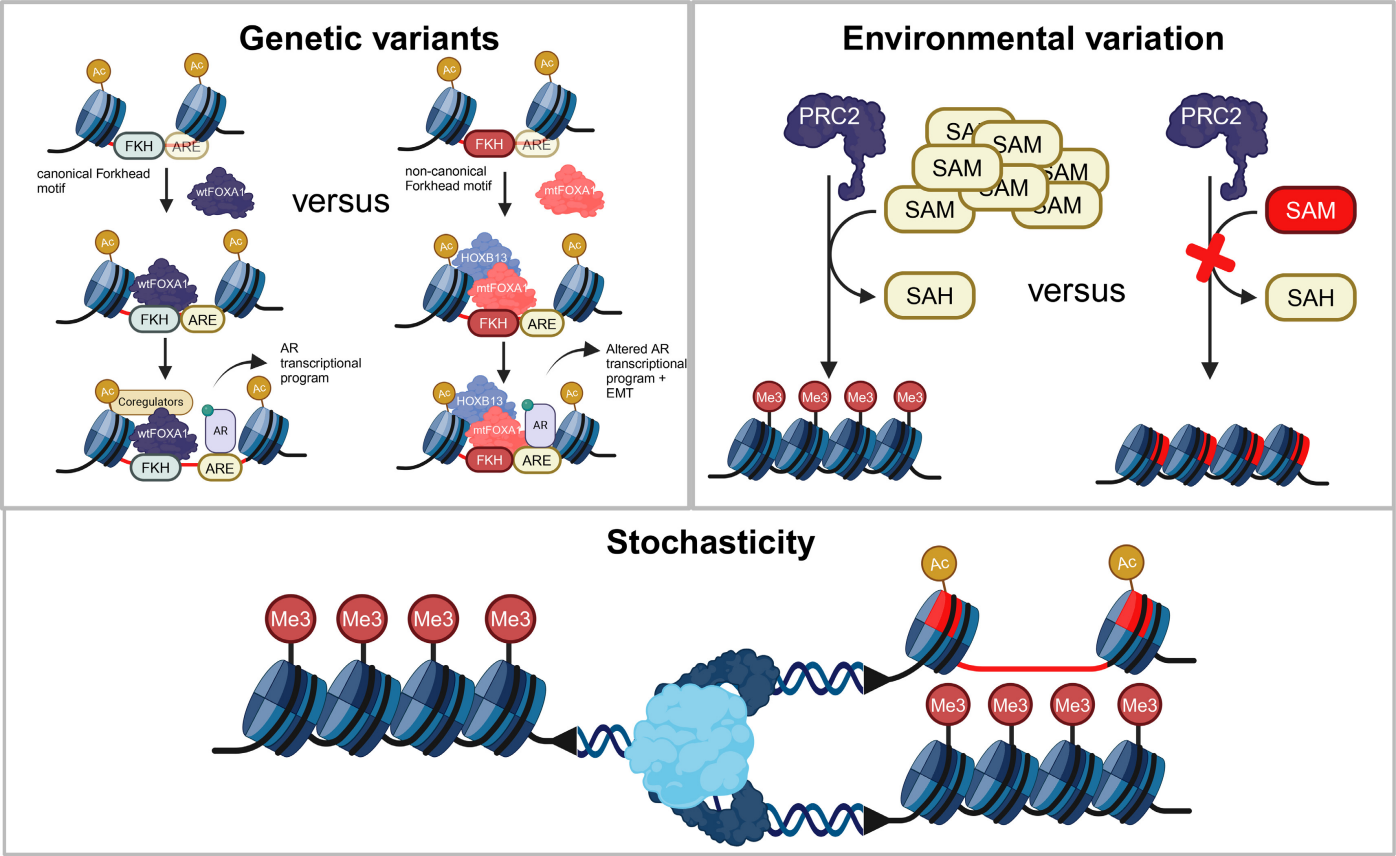
**Chromatin variants are not only caused by genetic variation.** *Genetic variants*: The top‐left section of the figure illustrates how genetic variation, particularly in chromatin remodelers like FOXA1, impact chromatin dynamics in prostate cancer. The wild‐type FOXA1 (wtFOXA1) interacts with the androgen receptor (AR) and co‐regulators at androgen response elements (ARE) on the chromatin, leading to normal transcriptional activation. However, the mutated FOXA1 (mtFOXA1) exhibits stronger interactions with the AR and alters the transcriptional program by engaging non‐canonical Forkhead motifs (FKH). This change promotes oncogenic transcriptional activities and enhances EMT, contributing to cancer progression. *Environmental variation*: The top‐right panel depicts how environmental factors, particularly those that alter the availability of key metabolites (e.g., SAM/SAH balance), can influence the activity of chromatin‐modifying complexes like PRC2. These environmental changes can lead to differential histone methylation patterns, thereby driving distinct epigenomic states that support cancer progression. The comparison between normal and disrupted SAM levels emphasizes the impact of metabolic disturbances on chromatin dynamics. *Stochasticity*: The bottom panel illustrates how random chromatin variants, such as erroneous histone modifications (e.g., methylation and acetylation), accumulate over time. These stochastic changes can lead to significant variations in chromatin states that impact gene expression, contributing to prostate cancer heterogeneity and progression. The image highlights how these random events might not be directed but can still significantly impact tumor evolution.

In the context of prostate cancer, three factors can influence the acquisition of chromatin variants—*genetic variants, environmental variation and stochasticity*. Each factor offers a different mechanism to engage cancer cells in an evolutionary process fuelled by epigenomic heterogeneity (see Figure 3). These factors are not mutually exclusive and may work in parallel to favor prostate cancer progression. Understanding the interplay between these factors can serve to develop more effective treatment strategies to hinder tumor evolution.

Genetic studies have identified hundreds of cancer driver genes across cancer types.^52^ The dominant gene ontology among prostate cancer driver genes centers on chromatin remodeling and chromatin organization. Many of these driver genes encode chromatin factors, including chromatin writers, erasers, readers, and remodelers.^17^, ^36^, ^53^ Recurrent alterations are observed in key chromatin remodelers like FOXA1 (see Figure 3; Genetic variants), RB1, and members of the SWI/SNF complex, including SMARCA1, ARID1A, and ARID2. Notably, the loss of CHD1, the most frequently altered chromatin remodeler in prostate cancer, occurring in approximately 15% of cases, leads to chromatin alterations that promote tumorigenesis.^17^, ^54^ Specifically, when CHD1 is lost, it results in a redistribution of the androgen receptor (AR) cistrome, creating a chromatin environment that favors oncogenic activity.^54^ This altered chromatin state, driven by the absence of CHD1, enables the AR to bind to new genomic regions that are associated with pro‐oncogenic pathways, thus contributing to tumor development.^54^ Therefore, the loss of CHD1 exemplifies how a genetic alteration can lead to chromatin changes that drive cancer progression. The analysis of different regions of primary tumors, metastases, and pre‐malignant lesions has demonstrated that the phylogenetic relationships determined by copy number diversity are closely reflected in DNA methylation patterns.^55^ This indicates that genetic and chromatin variants can emerge simultaneously and converge to drive similar oncogenic processes, leading to a unified evolution of the tumor.^35^, ^56^

Response to environmental variation, driven by chromatin dynamics, allows cells to switch between different states—developmental, regenerative, or pathological.^38^, ^57^ In tumors, such plasticity can arise from signals from the tumor microenvironment (TME) or therapeutic interventions.^58^ Metabolic fluctuations are key examples of environmental variations leading to the acquisition of chromatin variants because many metabolites serve as substrates or cofactors for chromatin factors.^59^ For instance, fluctuations in the level of S‐adenosylmethionine (SAM), the universal methyl donor, is linked to epigenetic reprogramming and castration resistance in prostate cancer.^60^, ^61^

Stochastic epigenomic variation refers to the random acquisition of chromatin variants in a cell. These changes can be either tolerated or may result in shifts to cellular function.^62^, ^63^, ^64^ DNA double‐strand breaks induction was suggested to be sufficient to start the acquisition of chromatin variants observed over aging.^65^ Prostate cancer undergoes significant genomic alterations as the disease progresses, exhibiting the highest levels of DNA damage reported as chromosomal instability (CIN) among all metastatic tumors.^66^ CIN is characterized by the presence of lagging chromosomes during anaphase, which often end up in micronuclei.^67^, ^68^, ^69^ Micronuclei exhibit distinct chromatin accessibility abnormalities, which can persist even after their genomic content is reintegrated into the nucleus.^67^ This process results in extensive epigenomic dysregulation and variability, driven by significant differences in histone post‐translational modifications and chromatin accessibility. Thus, CIN not only impacts genomic copy number but may also contribute to stochastic chromatin variant acquisition.

Gaining insight into how tumors develop and utilize epigenomic variation—whether through directed acquisition and stable inheritance of chromatin variants or through stochastic processes—is crucial for shaping therapeutic strategies. This understanding will inform the development of targeted epigenetic treatments, enhancing both preventive and anti‐cancer approaches.^40^

## 4 TECHNOLOGICAL ADVANCES FOR THE STUDY OF EPIGENOMIC HETEROGENEITY

Since 2009, advancements in single‐cell profiling technologies,^70^ such as single‐cell RNA sequencing (scRNA‐seq) and single‐cell Assay for Transposase‐Accessible Chromatin using sequencing (scATAC‐seq) have enabled the examination of gene expression and chromatin accessibility at the individual cell level, providing a deeper understanding of the heterogeneity within cell populations. For instance, by applying scATAC‐seq to 14,424 single cells from 18 prostate tumors, Eksi et al.^71^ discovered that high‐grade tumors lose shared chromatin features seen in low‐grade tumors but gain enrichment for certain transcription factor binding sites, indicating a shared trans‐regulatory program. Additionally, the study identified the genes NRXN1 and NLGN1, associated with neuronal adhesion, as highly accessible in high‐grade prostate cancer, offering valuable understanding into gene regulatory networks crucial for disease stratification.^71^ Similarly, Taavitsanien et al.^72^ used a combined approach of scATAC‐seq and scRNA‐seq to uncover chromatin and transcriptional changes linked to enzalutamide response and resistance in prostate cancer, revealing distinct chromatin landscapes and cell subpopulations that may predict treatment efficacy. Another study used single‐cell RNA sequencing to investigate cell variability in castration‐resistant prostate cancer (CRPC), uncovering increased inter‐cellular variability and a loss of lineage fidelity during the transition from luminal to basal phenotypes.^49^ These findings underscore the transformative potential of single‐cell technologies in advancing molecular diagnostics and personalizing cancer treatment by uncovering the chromatin variants driving tumor heterogeneity.

Emerging epigenomic technologies are revolutionizing our understanding of chromatin heterogeneity and tumor evolution, with profound implications for prostate cancer research. Single‐cell assays, including sort‐assisted chromatin immunocleavage (sortChIC)^73^ and scCUT&Tag,^74^ provide unparalleled resolution in profiling histone modifications and transcription factor occupancy. These technologies enhance both sensitivity and throughput, enabling precise mapping of active (e.g., H3K4me1, H3K4me3) and repressive (e.g., H3K27me3, H3K9me3) chromatin marks. For instance, sortChIC has uncovered critical epigenomic events such as lineage decisions during progenitor stages,^73^ while scCUT&Tag has overcome the limitations of bulk methods, uniquely capturing transcriptional regulation at the single‐cell level.^74^ Such approaches are particularly relevant for elucidating chromatin‐level mechanisms of cellular plasticity and therapy resistance in cancer.

Complementing these histone‐centric approaches, single‐cell DNA methylation (scDNAme) assays have transformed our understanding of epigenomic heterogeneity in tumor progression. Although bulk DNA methylation studies provided early insights,^75^ they lacked the resolution needed to track co‐occurrence of methylation patterns and their cellular origins. Techniques such as bisulfite treatment^62^ enabled more detailed analyses, leading to discoveries such as hypermethylation subtypes in advanced prostate cancer.^76^ Single‐cell methods like scRRBS and scWGBS^77^, ^78^ have advanced this field, allowing methylation profiling at unprecedented resolution and linking genomic alterations to distinct methylation signatures, as demonstrated with EGFR mutations in lung adenocarcinoma.^79^ Advanced methods such as scHi‐C^80^, ^81^ and parallel combination approaches like Methyl‐HiC^82^ further integrate insights into 3D chromatin interactions and methylome dynamics, offering a comprehensive view of gene regulation. These tools, when combined with histone modification and transcriptomic data,^40^ hold significant potential for dissecting prostate cancer heterogeneity and evolution.

Beyond single‐cell assays, spatial epigenomic technologies are opening transformative avenues for investigating chromatin states within the native tumor microenvironment. Techniques like spatial scATAC‐seq, employing solid‐phase barcode capture, enable spatially resolved profiling of chromatin accessibility across neighboring cell populations.^83^ Similarly, spatial histone modification quantification has delineated distinct enrichment patterns in breast tumors.^84^ These spatially integrated approaches are poised to uncover the interplay between chromatin and tumor ecosystems.

While these advancements have greatly enhanced our ability to measure epigenomic diversity within tumors and have proven effective on frozen tissues in some cancers, these methods typically focus on only one or two modalities rather than a full range of epigenomic assessments.^85^ Achieving a complete single‐cell and spatial resolution map of the tumor epigenome, including histone modifications, chromatin structure, DNA‐methylation and nucleosome positioning, remains challenging. Consequently, the field of single‐cell epigenomics in cancer is still developing, holding considerable promise for future discoveries in cancer research and treatment.

## 5 EPIGENOMIC VARIATION AND PROSTATE CANCER PROGRESSION

Prostate cancer presents a diverse clinical spectrum, with about 80% of men being diagnosed when the cancer is still confined to the prostate, 15% with locally advanced disease, and 5% with distant metastases.^3^ The prognosis for late‐stage metastatic prostate cancer is poor, with a 5‐year overall survival rate of just 30%.^3^ While certain prognostic factors like presence and quantity of bone and visceral metastases, ISUP grade, performance status, initial PSA and alkaline phosphatase are used in the clinical routine and offer some insight, current models lack molecular markers that could improve patient stratification and treatment personalization.^86^, ^87^, ^88^, ^89^

Chromatin variants are increasingly recognized as critical contributors to cancer development and progression. For instance, research on KRAS‐mutant pancreatic cancer models shows that inflammatory stress can lead to the establishment of chromatin states that are “primed” for cellular transformation.^90^ These states are characterized by enhanced deposition of H3K4me1 at genes associated with metaplasia, highlighting how inflammation can induce lasting epigenomic changes that predispose tissues to cancer.^91^, ^92^, ^93^ Similarly, in lung cancer models, chromatin alterations have been linked to metastatic progression, where these epigenomic changes promote the activation of malignant pathways.^94^

In prostate cancer, such mechanisms may play a critical role: In the development and progression of prostate cancer to metastatic castration‐resistant prostate cancer (mCRPC), transcription factors begin to interact with new DNA regulatory regions due to the emergence of chromatin variants. This reprogramming reactivates developmental pathways and contributes to cancer cell de‐differentiation.^26^, ^95^, ^96^, ^97^, ^98^, ^99^, ^100^ For example, the analysis of reactivated regulatory elements has led to the discovery and validation of previously unknown metastasis‐specific enhancers at key genes such as HOXB13, FOXA1, and NKX3‐1.^95^ This supports the idea that prostate adenocarcinoma cells don't create entirely new programs but instead repurpose previously lowly active or inactive ones.

Epigenetic reprogramming is further implicated in metastasis, with evidence showing that patients with regional lymph node metastasis or high‐grade tumors have significantly elevated expression of JAK–STAT signaling genes.^101^ This abnormal signaling is linked to the activation of an EMT lineage program, driving a metastatic phenotype. The role of JAK–STAT signaling and EMT highlights the potential for targeted therapies in aggressive prostate cancer. Additionally, alterations such as the loss of Zfp36 further contribute to aggressive cancer phenotypes by enhancing the proliferative and invasive capabilities of cancer cells.^102^ This loss leads to increased micro‐ and macro‐metastasis, with induced phenotypic plasticity enabling cancer cells to mimic immune cell characteristics, a process called lymphocyte mimicry.^103^ This mimicry, fueled by CIN and chronic activation of DNA sensing pathways like STING, promotes metastasis through an inflammatory response.^104^ Overall, chromatin‐based epigenetic modifications are key drivers of metastatic prostate cancer, contributing to aggressive phenotypes by promoting JAK–STAT signaling and EMT, as well as enabling cancer cells to adopt immune‐like characteristics.

Standard of care for patients with metastatic cancer involves systemic treatments, with local therapies used only as needed for symptom relief. The initial effectiveness of ADT in prostate cancer patients is based on its ability to target the dependency of hormone sensitive prostate cancer (HSPC) to the AR activity.^51^ However, prostate cancer cells can bypass the suppression effects of androgen ablation therapy by transforming intracellular steroid precursors into androgenic steroids.^105^, ^106^ To inhibit the re‐activation of AR‐promoted tumor growth next generation ARPIs are now available.^107^ Thus, combination therapy involving ADT and ARPI is for example employed to treat patients upon the detection of metastatic hormone‐sensitive recurrences (see Figure 1). However, metastatic HSPC (mHSPC) practically always progresses to mCRPC even under combination therapy, which presents as an incurable condition with limited life expectancy.

Recent research discovered that a subset of prostate cancer cells remains transcriptionally unaffected by the ARPI enzalutamide.^72^ Using scATAC and RNA sequencing, the study identified distinct intermediate cell states that emerge through alternative treatment pathways, driven partly by chromatin variant acquisition. Two key resistance‐associated prostate tumor states, PROSGenesis and Persist, were identified, which could predict outcomes in ARPI‐treated CRPC patients.^72^ These states also serve as prognostic markers for primary prostate cancer patients undergoing ADT.^72^ Interestingly, in treatment‐naïve patients, a high PROSGenesis score is associated with a longer response to ADT, likely due to the stronger influence of AR activity in these tumors. However, in general, high signature scores in these untreated patients are linked to a shorter time to progression, including biochemical recurrence.^72^

As clinical phenotyping of mCRPC has traditionally relied on morphologic and immunohistochemical analyses, with genomic landscapes of CRPC metastases within the same patient showing relative similarity,^108^ recent research has uncovered significant variations in chromatin accessibility in advanced prostate cancer, leading to the identification of therapy‐resistant mCRPC subtypes driven by specific transcription factors.^47^, ^51^ These mCRPC subtypes exhibit unique global chromatin accessibility profiles, particularly in relation to AR function, differing significantly from those seen in localized prostate cancer and benign prostate tissue. These findings suggest that personalized treatment approaches could be developed based on the unique epigenomic characteristics of each mCRPC subtype. Additionally, they also emphasize that phenotypic diversity in mCRPC is common.^109^

Cellular plasticity significantly contributes to the development of resistance to ARPIs in prostate cancer^110^, ^111^ and as many as 20% of advanced prostate cancers that are resistant to ARPI treatment show a loss of epithelial lineage identity, leading to reduced survival.^112^, ^113^, ^114^, ^115^, ^116^, ^117^ This process, known as lineage infidelity or trans‐differentiation, involves the transition of cells within a tissue from one identity to another and exemplifies cellular plasticity.^20^, ^118^, ^119^ Neuroendocrine prostate cancer (NEPC), which arises from epithelial tumors, is a well‐studied example of this phenomenon.^120^, ^121^, ^122^ This process involves the accumulation of chromatin variants leading prostate cancer cells to adopt an NEPC phenotype measured from changes in DNA methylation and chromatin accessibility.^51^

NEPC is histopathologically characterized by a loss of AR expression, minimal PSA secretion, and the expression of neuroendocrine markers such as chromogranins, synaptophysin (SYP), CD56, and neuron‐specific enolase (NSE).^113^, ^123^, ^124^ Although NEPC is largely treatment‐induced, rare de novo cases have been reported.^125^, ^126^ Current treatment regimes are similar to small cell lung cancer, a lung cancer that shares the neuroendocrine phenotype with NEPC,^127^ and therapies include platinum therapies with etoposide and taxanes.^128^ This subtype displays a highly aggressive clinical course with a median survival of less than a year.^129^

Even though the transformation to NEPC is facilitated by a combination of genetic and chromatin variations, key genetic events including mutations in TP53 and RB1, are essential but not sufficient alone for neuroendocrine transformation.^48^, ^130^, ^131^ EZH2, a crucial component of the Polycomb repressive complex 2 (PRC2), is essential for maintaining the transdifferentiated state,^42^, ^48^, ^131^ characterized by substantial changes in cistromes and interactions with specific protein partners and gene targets typically associated with the luminal epithelial phenotype.^132^ This transdifferentiation into NEPC is strongly influenced by an activated neuronal program,^133^ driven by key transcription factors such as ASCL1 and NEUROD1.^134^, ^135^, ^136^ These factors play a critical role in chromatin remodeling and modulating EZH2 activity, thereby promoting the development of a neuroendocrine phenotype, as revealed by lineage tracing studies.^48^, ^117^, ^131^ Notably, the progression to a NEPC phenotype appears to occur exclusively in vivo, likely because it depends on stromal or immune cell signals that are not present in models, such as organoid cultures.^49^

Targeting the epigenomic alterations that drives cellular plasticity offers a novel therapeutic approach for treating NEPC. EZH2 inhibitors, for instance, have shown potential in reversing neuroendocrine transformation and restoring sensitivity to AR‐targeted therapies.^48^, ^137^ Similarly, LSD1 inhibitors have the potential to reverse neuroendocrine phenotypes in prostate cancer.^138^, ^139^ The effectiveness of these strategies underscores the need for molecular diagnostic assays to guide patient selection and optimize treatment timing.

The transition to NEPC represents a significant challenge in the management of advanced prostate cancer. Understanding the chromatin‐based mechanisms driving this lineage plasticity is crucial for developing targeted therapies. Future research should focus on further elucidating the role of chromatin factors in this process, as well as optimizing therapeutic strategies that exploit epigenomic vulnerabilities. Molecular stratification of patients based on their chromatin variants will be essential for the successful implementation of these therapies in clinical practice.

## 6 LEVERAGING CHROMATIN VARIANTS FOR PRECISION ONCOLOGY

Genome‐wide sequencing has revolutionized the molecular analysis of cancer, making it more cost‐effective and accessible while transforming the treatment landscape for advanced prostate cancer.^140^, ^141^ Techniques such as whole‐genome sequencing (WGS), whole‐exome sequencing (WES), and targeted sequencing have become indispensable tools in this field.^142^ Targeted sequencing, in particular, offers the advantage of deep coverage in specific regions of interest but carries the risk of missing crucial driver mutations. This limitation may be addressed by designing patient‐specific panels tailored to individual tumors, although this approach is not yet widely feasible and has its pitfalls.^143^ The challenge lies in balancing the need for higher coverage in targeted areas with the benefits of a broader but less detailed approach.^142^ These advancements have shifted prostate cancer treatment from a one‐size‐fits‐all model to a more personalized approach, integrating targeted therapies guided by genomic insights^141^, ^144^ (see Box 1 and Table 1).

**TABLE 1:** Current targeted therapies in the prostate cancer landscape.

Drug	Disease State	Mutations	Results	Reference	Comment	Approval FDA	Approval EMA
*PARP inhibitors*							
Olaparib	2nd line mCRPC (after Enzalutamide or Abiraterone)	BRCA1/2, ATM	rPFS for Olaparib and OS in interims analysis (not significant)	PMID: 32343890		Deleterious germline or somatic homologous recombination repair (HRR) gene‐mutated metastatic castration‐resistant prostate cancer (mCRPC), who have progressed following prior treatment with enzalutamide or abiraterone	mCRPC with BRCA1/2 mutation, after treatment with other prostate cancer medicines, including a new hormonal agent; unselected mCRPC patients in whom chemotherapy is not clinically indicated
Rucaparib	From 3rd line mCRPC (who had been treated with androgen receptor‐directed therapy and taxane‐based chemotherapy)	BRCA‐mutated (germline and/or somatic)	ORR by IRR was 46% (37/81)	PMID: 37277275		Pretreated mCRPC with BRCA1/2 mutations	No approval
*PARP inhibitor combination*							
Olaparib + Abiraterone	1st line mCRPC	Unselected	rPFS regardless of mutation status	PMID: 37714168	Was driven by the benefit of patients with BRCA1/2 mutations	BRCA‐mutated mCRPC PMID: 38127780	mCRPC with BRCA1/2 mutation, after treatment with other prostate cancer medicines, including a new hormonal agent; unselected mCRPC patients in whom chemotherapy is not clinically indicated
Talazoparib + Enzalutamide	1st line mCRPC	HRR	rPFS	PMID: 37285865, PMID: 37882449 (NCT04821622)	Was driven by the benefit of patients with BRCA1/2 mutations; confirmed in a large cohort with patients with HRR mutations PMID: 38049622	HRR gene‐mutated mCRPC PMID: 38452327	Unselected mCRPC patients in whom chemotherapy is not clinically indicated
Niraparib + Abiraterone	1st line mCRPC	HRR gene‐mutated mCRPC	rPFS for BRCAm patients with a median of 16.6 months vs. 10.9 months (HR 0.53; 95% CI 0.36, 0.79; *p* = .0014)	PMID: 36952634		Deleterious or suspected deleterious BRCA‐mutated mCRPC	mCRPC patients with a BRCA1/2 mutation
Niraparib + Abiraterone	mHSPC	HRR		NCT04497844			
Saruparib (AZD5305) + physician's choics of ARPI (Abiraterone, Darolutamide, Enzalutamide)	mHSPC	HRR		NCT06120491			
*Radioligand therapy*							
Lu177‐PSMA‐RLT	mCRPC (after 1 ARPI + taxane based chemotherapy)		PSA responses rate (66% vs. 37% by intention to treat; and 66% vs. 44% by treatment received)	PMID: 33581798	Many more studies in earlier settings or combination therapies under investigation PMID: 36806966		
*Immunotherapy*							
Pembrolizumab	Solid tumors	Mutations in the dMMR region or evidence of MSI and/or TMB > 10 Mut/Mb	ORR was 39.6% among 149 patients with 15 different tumor types (95% confidence interval, 31.7–47.9)	PMID: 30787022, PMID: 35357449	Affecting approximately 1%–2% of all prostate cancer patients; without other treatment options; retrospective data do support the use of immunotherapy in prostate cancer with these genetic characteristics	Yes, agnostic	No approval
*AKT inhibitors*							
Ipatasertib + Abiraterone	mCRPC		PTEN loss: extended PFS 18.5 vs. 16.5 months, HR 0.77, *p* = .034; no difference was observed in the intention‐to‐treat population and no OS benefit in the PTEN loss group	PMID: 34246347			
Capivasertib + Docetaxel	mCRPC		OS of 25.3 months compared to 20.3 months (HR 0.70, 95% CI 0.47–1.05; *p* = .09).	PMID: 33326257, PMID: 35688662	Independent of the presence of a PI3K/AKT/PTEN alteration		
Capivasertib + Abiraterone	de novo mHSPC	PTEN loss		NCT04493853			
*FGFR‐inhibitors*							
Erdafitinib	Multiple solid tumors	FGFR‐alterations	No response to this specific targeted therapy was observed in the 2 patients with prostate cancer	PMID: 37541273			
*MEK inhibitor*							
Trametinib	mCRPC	BRAF fusion		PMID: 35994225	Case report		

*Note*: References: PMID: 32343890,^170^ PMID: 37277275,^179^ PMID: 37714168,^180^ PMID: 38127780,^181^ PMID: 37285865, PMID: 37882449,^182^, ^183^ PMID: 38049622,^184^ PMID: 38452327,^185^ PMID: 36952634,^186^ NCT04821622,^187^ NCT06120491,^188^ PMID: 33581798,^171^ PMID: 36806966,^172^ PMID: 30787022, PMID: 35357449,^173^, ^174^ PMID: 34246347,^175^ PMID: 33326257, PMID: 35688662,^189^, ^190^ NCT04493853,^191^ PMID: 37541273,^177^ PMID: 35994225.^178^

In prostate cancer management, recognizing cellular plasticity can be particularly challenging due to its dynamic nature at the cellular level. This plasticity is most noticeable when a primary prostate tumor or metastatic lesion exhibits mixed histology or a shift in phenotype from the original tumor. For example, in cases where there is a transformation to NEPC, targeted genomic assays like exome sequencing can identify new genetic alterations related to this plasticity or resistance to treatment, such as the loss of RB1 or TP53.^145^, ^146^ Immunohistochemistry (IHC) can confirm these changes, but in both cases obtaining new biopsies can be difficult and carries risk. Thus, there is a pressing need for early detection methods to identify patients at risk for cellular plasticity and to develop targeted treatments that address these evolving characteristics.

Parallel to these developments, the reversible nature of chromatin variants through chemical agents has sparked significant interest in epigenetic therapies. As the mechanisms behind these processes are increasingly understood, therapeutic agents that can modify their function have emerged.^147^ Currently, the U.S. Food & Drug Administration (FDA) has approved three classes of epigenetic inhibitors: DNA methyltransferase (DNMT) inhibitors, histone deacetylase (HDAC) inhibitors, and enhancer of zeste homolog 2 (EZH2) inhibitors. For example, EZH2 inhibitors have been shown to re‐sensitize resistant prostate cancer cells by reducing repressive histone methylation, which restores the expression of tumor‐suppressive genes and re‐establishes sensitivity to ARPIs.^48^ These modulators have profoundly deepened our understanding of how cancer cells leverage chromatin variants for growth and survival.^40^, ^147^ Clinical trials are ongoing to evaluate the use of epigenetic therapies in combination with other drugs, aiming to improve responses to standard treatments in prostate cancer (see Table 2). In the coming years, we foresee a substantial increase in studies leveraging temporal epigenomic data across diverse assays to elucidate the mechanisms driving tumor evolution. A particularly promising development is the integration of spatial omics technologies with lineage barcoding approaches,^148^, ^149^ which enable the tracing of cellular ancestry and dynamic changes in tumor architecture over time.

**TABLE 2:** Summary of ongoing and completed clinical trials for epigenetic drugs in prostate cancer (adapted from^147^).

Drug	Disease state	In combination with	Study number	Phase	Status	Reason for withdrawal/ termination	Reference articles (PMID)
*HDAC inhibitors*							
Pracinostat (SB939)	CRPC		NCT01075308	Phase 2	Completed		25,983,041
*BET inhibitors*							
AZD5153	Malignant solid tumors (prostate, ovarian, breast, pancreatic) and lymphoma	Olaparib +/−	NCT03205176	Phase 1	Completed		
Mivebresib (ABBV‐075)	Prostate cancer, Breast cancer, NSCLC, AML, MM, SCLC, NHL		NCT02391480	Phase 1	Completed		33,934,351
Molibresib (GSK525762)	CRPC, NMC, SCLC, NSCLC, CRC, neuroblastoma, breast cancer, MYCN‐driven solid tumors		NCT01587703	Phase 1	Completed		34,724,226
	CRPC	Androgen deprivation	NCT03150056	Phase 1	Terminated	Protocol‐defined‐futility	
Birabresib (MK‐8628/OTX015)	CRPC, NMC, breast cancer, NSCLC		NCT02698176	Phase 1	Terminated	Limited efficacy	
	CRPC, NMC, breast cancer, NSCLC, pancreatic cancer		NCT02259114	Phase 1	Completed		
ZEN‐3694	Metastatic CRPC	Enzalutamide, pembrolizumab	NCT04471974	Phase 2	Recruiting		
	CRPC	Enzalutamide	NCT04986423	Phase 2b	Recruiting		
	CRPC		NCT02705469	Phase 1	Completed		
	CRPC	Enzalutamide	NCT02711956	Phase 1b/2a	Completed		32,694,156
*EZH2 inhibitors*							
PF‐06821497	CRPC, SCLC, follicular lymphoma		NCT03460977	Phase 1	Recruiting		
SHR2554	CRPC	SHR3680	NCT03741712	Phase 1/2	Terminated	Sponsor decision	
CPI‐1205	CRPC		NCT03480646	Phase 1/2	Unknown		
Valemetostat (DS‐3201b)	Prostate, urothelial and renal cell carcinomas	Ipilimumab	NCT04388852	Phase 1	Recruiting		
*EED inhibitor*							
ORIC‐944	Metastatic Prostate Cancer		NCT05413421	Phase 1	Recruiting		
*p300‐CBP inhibitors*							
FT‐7051	CRPC		NCT04575766	Phase 1	Terminated	Sponsor decision	
Inobrodib (CCS1477)	CRPC, NSCLC, breast cancer, advanced solid tumors	Alone or in combination with select cancer‐specific drug regimens	NCT03568656	Phase 1/2a	Recruiting		
EP31670/NEO2734	CRPC, NMC		NCT05488548	Phase 1	Recruiting		
*KAT6A inhibitor*							
PF‐07248144	CRPC, ER + Her2‐ breast cancer, NSCLC	Fulvestrant, letrozole, palbociclib	NCT04606446	Phase 1	Recruiting		
**LSD1 inhibitors**							
CC‐90011	Prostate cancer	Abiraterone + Prednisolon	NCT04628988	Phase 1	Completed		
Iadademstat (ORY‐1001)	Prostate cancer (neuroendocrine), SCLC				to begin shortly		

*Note*: PMID: 25983041,^192^ PMID: 33934351,^193^ PMID: 34724226,^194^ PMID: 32694156.^195^

Advancements in molecular profiling have revolutionized personalized cancer treatment by enabling the precise selection of therapies, real‐time assessment of treatment responses, early detection of drug resistance, and effective monitoring of tumor relapse.^141^, ^144^ Traditionally, tumor profiling relies on invasive surgical biopsies, which pose several challenges, including the difficulty of acquiring adequate, high‐quality samples and the limitations of performing repeated biopsies for dynamic tumor monitoring.^142^, ^150^, ^151^ To address these challenges, oncology research has increasingly pivoted toward less invasive methods like liquid biopsies, which provide a more practical and comprehensive approach to capturing tumor dynamics and heterogeneity over time.

Liquid biopsy technologies are emerging as valuable tools for non‐invasive cancer diagnosis and monitoring.^142^, ^150^, ^152^ Techniques that analyze circulating tumor DNA (ctDNA), circulating tumor cells (CTCs), exosomes, and cell‐free DNA/RNA (cfDNA/cfRNA) offer insights into tumor genetics and epigenetics.^142^ While the analysis of ctDNA to detect mutation and copy‐number alterations is already incorporated in the clinical routine for targeted therapy,^116^, ^145^, ^153^, ^154^, ^155^, ^156^, ^157^, ^158^ plasma‐based epigenomic profiling, for instance, has revealed chromatin variants associated with treatment resistance and cancer progression, providing a potential alternative to traditional tissue biopsies.

The detection and management of early‐stage prostate cancer face significant challenges due to the lack of consensus genetic mutations. For example, alterations in the *SPOP* gene, among the most frequent in early prostate cancer, occur in only 8%–13% of cases, highlighting the limitations of genetic approaches in early diagnostics.^23^, ^159^ Consequently, alterations detected through urine‐based approaches offer a promising non‐invasive strategy for identifying early disease states, potentially reducing the need for invasive prostate biopsies. A notable example is the Epigenetic Cancer of the Prostate Test in Urine (epiCaPture).^160^ This multi‐biomarker panel quantitatively measures DNA hypermethylation in urine samples at the regulatory regions of six genes (*GSTP1, SFRP2, IGFBP3, IGFBP7, APC*, and *PTGS2*), all implicated in prostate carcinogenesis. This approach shows great potential when used in combination with existing tools such as PSA testing and risk calculators. By guiding biopsy decisions, epiCaPture can help alleviate patient harm and reduce the burden on healthcare systems caused by over‐performing biopsies.^160^

As the epigenome shows promise for distinguishing phenotypes, assays to characterize chromatin variants from the bloodstream were developed. Most cfDNA in the bloodstream is associated with nucleosomes when it is released from dying cells.^161^, ^162^, ^163^ This results in DNA fragmentation that reflects the nonrandom cleavage by nucleases. New methods for analyzing cfDNA fragmentation patterns from plasma in cancer research can be conducted using standard WGS techniques.^164^, ^165^, ^166^, ^167^, ^168^, ^169^ A study by Baca et al.^152^ demonstrates that cell‐free chromatin immunoprecipitation (cfChIP) for H3K27ac detected the activation of an enhancer of the AR gene, which contributes to castration resistance in prostate cancer—an alteration that DNA methylation analysis could not detect due to hypomethylation at the locus.^152^

Another recent study identified key transcriptional regulators associated with specific cancer phenotypes, such as AR, ASCL1, HOXB13, HNF4G, and GATA2, from ctDNA.^163^ They developed predictive models for distinguishing NEPC from androgen receptor‐positive prostate cancer (ARPC) in plasma samples, achieving 97% accuracy for dominant phenotypes and 87% for mixed phenotypes. This approach demonstrates that ctDNA analysis offers diagnostic capabilities comparable to traditional methods like IHC and transcriptome profiling from tumor biopsies, with potential advantages for precision oncology.^163^ Additionally, the technique detected elevated H3K27ac signals at specific binding sites in plasma from patients with treatment‐induced NEPC, a transformation that genetic assays cannot detect.^152^ This method enables the detection of chromatin accessibility changes and transcriptional programs associated with different cancer phenotypes, including neuroendocrine differentiation in prostate cancer.

## 7 CONCLUSION

The integration of chromatin variant analysis with liquid biopsy technologies represents a significant leap forward in the field of precision oncology. By concentrating on the epigenomic landscape and utilizing non‐invasive diagnostic tools, researchers and clinicians can tailor more effective and personalized cancer treatments covering heterogeneity and plasticity. However, to advance research and precision oncology, a multi‐disciplinary approach involving close collaboration between clinicians, researchers, and the pharmaceutical industry is paramount. This collaboration should focus on implementing comprehensive multi‐omics strategies that include primary multi‐focal biopsies, matched biopsies of metastatic sites, and serial blood draws at critical clinical junctures. These key time points should include baseline assessments at diagnosis, post‐treatment evaluations, during PSA rise, at phenotypic transitions (such as the switch from hormone‐sensitive to castration‐resistant prostate cancer), and upon relapse or the emergence of therapy resistance. Such an approach would enable a more detailed understanding of tumor evolution, resistance mechanisms, and the timing of epigenetic and molecular changes. By integrating these data, the field can move towards more precise, targeted therapies that are applied at the optimal time, thereby improving patient outcomes and addressing the complex biology of prostate cancer.

## AUTHOR CONTRIBUTIONS

**Kira Furlano:** Conceptualization; visualization; project administration; writing – original draft; writing – review and editing. **Tina Keshavarzian:** Writing – review and editing. **Nadine Biernath:** Writing – review and editing. **Annika Fendler:** Writing – review and editing; resources. **Maria de Santis:** Writing – review and editing; resources; visualization. **Joachim Weischenfeldt:** Writing – review and editing; resources. **Mathieu Lupien:** Conceptualization; writing – original draft; writing – review and editing; resources; supervision; visualization; project administration.

## CONFLICT OF INTEREST STATEMENT

All authors declare that they have no conflict of interest.

BOX 1 **Advances and challenges in the clinical management of advanced prostate cancer: The role of targeted therapies**

Over the past two decades, the treatment landscape for advanced prostate cancer has evolved significantly from a one‐size‐fits‐all approach to a more tailored strategy that includes a variety of new therapeutic options. These include second‐generation anti‐androgens (ARPIs, chemotherapy, PARP inhibitors, immunotherapy, and radiopharmaceuticals, all of which have collectively improved patient outcomes; see Figure 1). Despite these advancements, significant gaps remain in the management of metastatic prostate cancer.

Modern treatments now often involve the use of precision oncology, particularly for a subset of metastatic prostate cancer cases where genomic testing has become a key tool. PARP inhibitors, the most studied class of targeted therapies, have shown benefits in mCRPC patients with BRCA1/2 or ATM mutations, with Olaparib demonstrating efficacy after one line of ARPI in the PROFOUND study.^170^ Another promising targeted therapy is Lutetium‐177 radioligand therapy, which targets the prostate‐specific membrane antigen (PSMA) and has shown a 66% PSA response rate post‐ARPI and taxane‐based chemotherapy,^171^ so that studies are investigating earlier‐stage use and combinations with Olaparib.^172^ Approximately 1%–2% of prostate cancer patients with deficient mismatch repair (dMMR) mutations, microsatellite instability (MSI), or high tumor mutational burden (TMB) (>10 Mut/Mb) may benefit from the FDA‐approved, tumor‐agnostic treatment Pembrolizumab.^173^, ^174^ Additionally, AKT inhibitors like Ipatasertib have shown progression free survival (PFS) but no overall survival improvement (OS) in mCRPC patients with PTEN loss.^175^, ^176^ Other targeted therapies like Fibroblast growth factor receptor (FGFR) inhibitors have shown limited response,^177^ though MEK inhibitors present promising case reports^178^ (see Table 1).

However, the rarity of certain mutations and the lack of mature comparative data highlight the need for early, repeated genetic sequencing and large‐scale studies. These challenges underscore the multi‐dimensional tumor phenotypic heterogeneity in prostate cancer, necessitating even more precise and personalized approaches to treatment. As we advance, the integration of epigenetic therapies that target chromatin variants and reprogram the epigenome will be crucial in addressing the complexity of prostate cancer and enhancing the effectiveness of precision oncology.
